# 3D printed bone models in oral and cranio-maxillofacial surgery: a systematic review

**DOI:** 10.1186/s41205-020-00082-5

**Published:** 2020-10-20

**Authors:** Matteo Meglioli, Adrien Naveau, Guido Maria Macaluso, Sylvain Catros

**Affiliations:** 1 University Center of Dentistry, Department of Medicine and Surgery, University of Parma, Via Gramsci 14, 43126 Parma, Italy; 2 Department of Prosthodontics, Dental Science Faculty, University of Bordeaux, 46 rue Léo-Saignat, 33076 Bordeaux, France; 3 Dental and Periodontal Rehabilitation Unit, Saint Andre Hospital, Bordeaux University Hospital, 46 rue Léo-Saignat, 33076 Bordeaux, France; 4 Biotis Laboratory, Inserm U1026, University of Bordeaux, 46 rue Léo-Saignat, 33076 Bordeaux, France; 5 IMEM-CNR, Parco Area delle Scienze 37/A, 43124 Parma, Italy; 6 Department of Oral Surgery, UFR d’Odontologie, University of Bordeaux, 46 rue Léo-Saignat, 33076 Bordeaux, France; 7 Service de Chirurgie Orale, CHU de Bordeaux, 46 rue Léo-Saignat, 33076 Bordeaux, France

**Keywords:** 3D printing, Additive manufacturing, Bone model, Surgical training, Preoperative planning, Simulation

## Abstract

**Aim:**

This systematic review aimed to evaluate the use of three-dimensional (3D) printed bone models for training, simulating and/or planning interventions in oral and cranio-maxillofacial surgery.

**Materials and methods:**

A systematic search was conducted using PubMed® and SCOPUS® databases, up to March 10, 2019, by following the Preferred Reporting Items for Systematic reviews and Meta-Analysis (PRISMA) protocol. Study selection, quality assessment (modified Critical Appraisal Skills Program tool) and data extraction were performed by two independent reviewers. All original full papers written in English/French/Italian and dealing with the fabrication of 3D printed models of head bone structures, designed from 3D radiological data were included.

Multiple parameters and data were investigated, such as author’s purpose, data acquisition systems, printing technologies and materials, accuracy, haptic feedback, variations in treatment time, differences in clinical outcomes, costs, production time and cost-effectiveness.

**Results:**

Among the 1157 retrieved abstracts, only 69 met the inclusion criteria. 3D printed bone models were mainly used as training or simulation models for tumor removal, or bone reconstruction. Material jetting printers showed best performance but the highest cost. Stereolithographic, laser sintering and binder jetting printers allowed to create accurate models with adequate haptic feedback. The cheap fused deposition modeling printers exhibited satisfactory results for creating training models.

**Conclusion:**

Patient-specific 3D printed models are known to be useful surgical and educational tools. Faced with the large diversity of software, printing technologies and materials, the clinical team should invest in a 3D printer specifically adapted to the final application.

## Introduction

Technological development strongly drives the evolution of oral and cranio-maxillofacial surgery [1]. Among all the additive manufacturing (AM) processes, “three-dimensional printing” (3DP), often used synonymously with additive manufacturing, is playing an ever-growing role. This technology involves the fabrication of objects through the deposition of material using a print head, nozzle, or other printing technology [2]. It allows creating objects layer-by-layer through computer-aided design/computer-aided manufacturing (CAD/CAM). It was originally developed in the 1980s to accelerate the production of small and custom-designed objects, but it revolutionized the prototyping concepts and embraced many applications in manufacturing industries. Later on, AM’s applications started to be integrated in several medical techniques and procedures, giving some important inputs to various domains, such as dentistry, maxillofacial surgery, orthopedics and neurosurgery. Frequent clinical applications of 3D printing in everyday practice include the fabrication of surgical templates employed to improve the accuracy of the surgery and reduce the duration and morbidity of surgical interventions. It is now applied in routine in oral and craniofacial surgery [3, 4]. More recently, the progress made in the 3D-printing of implantable biomaterials were applied to the fabrication of custom implants, based on patients’ radiological data: even if a large amount of these commercial custom implants are milled, 3D-printing technologies can be employed for large bone defects reconstruction like cranioplasty [5] or Temporo-mandibular joint replacement [6].

**Fig. 1:**
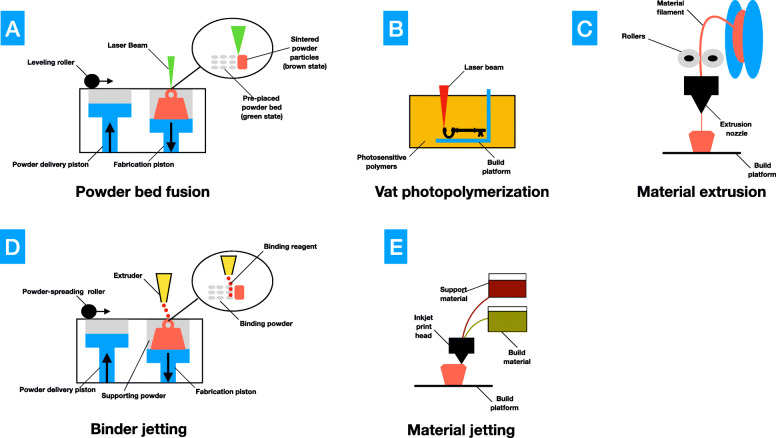
Schematic representation of rapid prototyping techniques for surgery applications: (**a**) Powder bed fusion (**b**) Vat photopolymeration (**c**) Material extrusion (**d**) Binder jetting (**e**) Material jetting

**Fig. 2:**
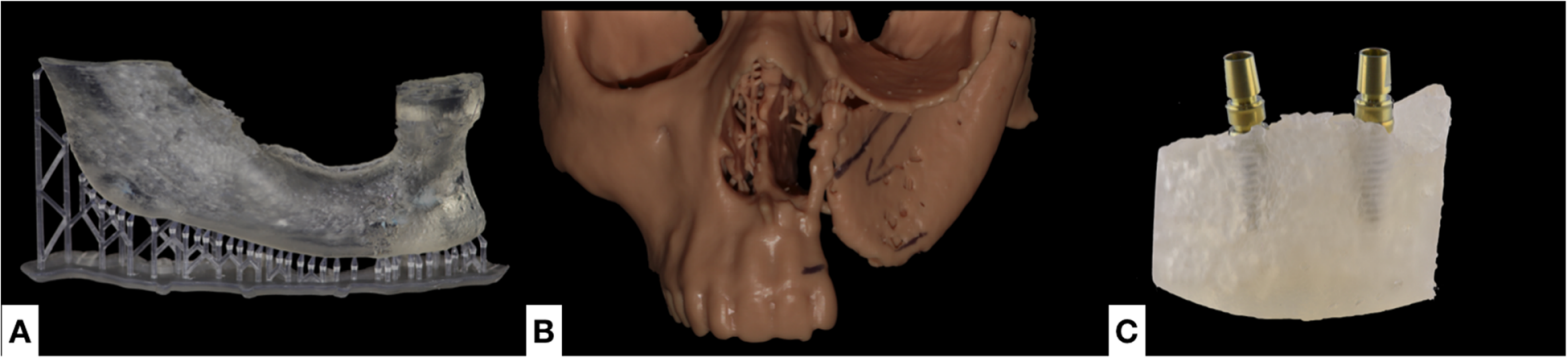
Examples of anatomical models fabricated by Additive manufacturing. A: A mandible model fabricated using SLA to serve as a template for bone allograft preparation during surgery. B: A model fabricated using SLA to visualize the extent of a bone defect (cleft) for planning the bone reconstructive surgery. C: A model representing a section of a mandible. It was used for training students in dental implant surgery

Three-dimensional printing techniques involve creation of accurate physical 3D models from the patient’s radiological data. The first step consists in obtaining the Digital Imaging and COmmunications in Medicine (DICOM) files from patient’s imaging exams, such as computed tomography (CT) or magnetic resonance imaging (MRI) scans. Then software is used to transform them into a digital 3D object file, such as standard tessellation language (STL), among other formats. For surgical model fabrication, this new file can be printed with different techniques, such as vat photopolymerization (VP), material extrusion (ME) or binder jetting (BJ). 3D printing encompasses different techniques, each of them having its own benefits and drawbacks (Fig. 1). Several printing materials can be used, each with specific mechanical and accuracy properties. Sometimes, a post-curing step is required to obtain the finished product [7]. The obtained surgical models can fulfill three different purposes: training, planning and simulating. An example of three different models is shown in Fig. 2.

A training model is produced to enhance the quality of the teaching, by allowing students and clinicians to perform high-quality surgical training. Adequate haptic feedback and moderate cost are the most important characteristic of training models. Instead of training on cadaver or animal models, students can enhance their skills on accurate 3DP models that reproduce the haptic feedback of the patient’s bone [8]. Although cadaveric specimens have high anatomical and physical validity, they are often challenging to obtain, lack patient-specific pathologic features, and are associated with costs that may be prohibitive to repetitive training [9]. Several authors described temporal bone surgeries [10, 11], implant treatment or maxillary sinus floor augmentation [12] training in realistic in vitro conditions using these 3D-printed models. Thus, one of the most important characteristics of a training model is the low cost.

Secondly, a 3DP model could be made for planning complex surgeries and to fully understand the patient’s conditions. The manipulation of the patient anatomical structures helps to get a better understanding of his specific condition and to plan the required surgical interventions [13]. These models are often used in orthognathic and reconstructive surgeries, giving the possibility of a better comprehension and more predictable results [14–17]. Measurements and surgical pre-operative evaluations can be performed on these models. Thus, the most important characteristic of a planning model is accuracy.

Finally, a simulating model is produced to perform a surgery before it is done in clinics. This type of models must be accurate with a patient-like haptic feedback. Users can act on these models like they are working on patients. Simulating models are used by clinicians before preforming important surgical interventions, such as tumor excisions [18] and pediatric mastoidectomy [19]. The right mechanical properties, such as the elastic modulus, the stiffness or the drilling force, are fundamental parameters that allow the operator to experience haptic feedback similar to that encountered during actual surgery. Thus, one of the most important characteristic of a simulating model is related to its mechanical properties.

A surgeon wanting to invest in a printer can be overwhelmed in his choice by the numerous options available, between printing materials and technologies. Moreover, the cost-benefit ratio must be taken into consideration, as cheap technologies can be sufficient for some applications. Our hypothesis was that each application of the surgical model could be correlated with a specific 3D printing technology. This systematic review investigated the state of the art of printing materials and techniques proposed to create models for training, simulating and planning interventions in oral and craniofacial surgery. The research focused on the regions of head and neck, scanning articles that belong to different fields: dentistry and oral surgery, maxillofacial surgery, ear-nose-throat surgery and cranial surgery. These results also provided practical suggestions for choosing the optimal 3D printing technique and material for each application objective.

## Methods

### Study protocol

The protocol of this systematic review was registered in ‘PROSPERO: International prospective register of systematic reviews’ (CRD42019117468) and followed the PRISMA guidelines.

The main question, that was not PICO compliant, was: ‘What are the existing printed bone models currently used for training, planning and simulating interventions in oral and cranio-maxillofacial surgery?’ The impossibility of using a PICO question and performing a meta-analysis are two missing points of PRISMA checklist.

### Search strategy

Medline (PubMed) database and Scopus database were searched up to March 10, 2019 with the following equation:

*(additive manufacturing OR rapid prototyping OR bone model OR bone models OR anatomical models OR anatomical model OR phantom OR phantoms OR simulation model OR simulation models OR 3D−printed models OR 3D printed models OR 3D−printed model OR 3D printed model) AND (planning OR hands−on OR train OR training OR simulation) AND (surgery OR surgical OR dentistry OR dental OR teaching OR pre−operative) AND (maxillofacial OR oral OR skull base OR jaw bones OR jaw OR sinus OR mandible OR temporal bone OR teeth OR maxilla OR human bone OR implant) NOT biology NOT cartilage NOT mathematical*

This process only selected articles that had search terms in the title or in the abstract without any restriction on language.

The search was also launched with the following MeSH (PubMed) terms: *(“Surgical Procedures, Operative” OR “Teaching”) AND “Printing, Three-Dimensional” AND “Models, Anatomic” AND “Head”.*

Other interesting original research articles were added through manual search.

### Science mapping

A science mapping analysis of subject domains was performed by using keyword co-occurrence networking on VOSviewer (free software, version 1.6.15, Centre for Science and Technology Studies, Leiden University, The Netherlands, 2017). A network analysis of the Pubmed MeSH keywords was generated from the matrix of retrieved papers (threshold value at 90). The MeSH terms-document matrix allowed to measure document similarities between clusters of topics.

### Study selection

All original full papers written in English/French/Italian and dealing with the fabrication of 3D printed models of head bone structures obtained from 3D imaging data were considered as potentially eligible. Case report, case series, pilot studies and comparative studies were included in this research.

Literature reviews, conference abstracts, articles employing animal tissues or cadaver models or models not aimed to the head region were excluded.

### Study analysis

All the retrieved references, after launching the search algorithm, were managed using Endnote® Abstracts of studies retrieved using the search strategy and those from additional sources were screened independently by three authors (M.M, A.N. and S.C) to identify studies that potentially met the inclusion criteria. Papers fulfilling the inclusion criteria, and those presenting insufficient data in the title and the abstract to make a decision, were selected for full analysis. After reading the full texts, the proper articles were included in an evidence table. Any disagreement over the eligibility of studies was resolved through discussion and consensus among the authors.

### Quality assessment

The quality of the included studies was assessed using a modified version of the Critical Appraisal Skills Programme (CASP) tool [20]. For each of the 10 questions of this tool, there were three possible answers: ‘Yes’, ‘Can’t tell’ or ‘No’. Every ‘Yes’ scored 1 point, while ‘No’ or ‘Can’t tell’ scored 0 points. Total scores were converted to percentages and studies were allocated to one of three categories; ‘Good quality’ for a score of 67–100%, ‘Average quality’ for 34–66% and ‘Poor quality’ for 0–33%.

### Data extraction

The data were extracted and critically appraised by two independent authors (M.M and G.M.M.).

Using a standardized data extraction form, the authors extracted the following data: year of publication, data acquisition system, type of printing technique involved, material, accuracy, production time, haptic feedback, treatment time, clinical outcomes, cost and purpose of the publication: training, planning, simulation (multiple possibilities for each paper).

### Data analysis

A narrative synthesis of the data was conducted due to the heterogeneity of study designs and methods. For the same reason it was not possible to perform a meta-analysis.

In order to address the general question of defining the state of the art of 3D printing to address the surgical planning, simulation and training needs, the authors identified as crucial these characteristics: accuracy, haptic feedback and cost.

## Results

### Search general results

**Fig. 3:**
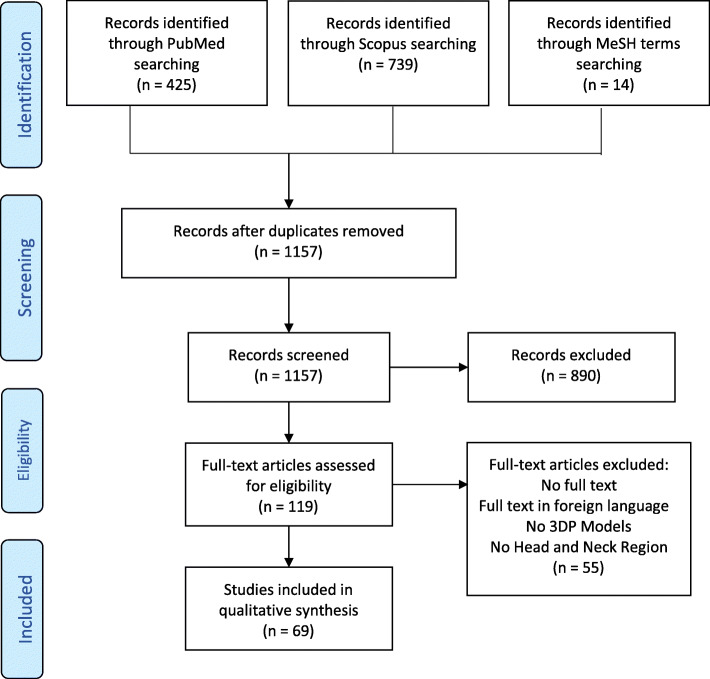
PRISMA flow chart

After database screening and removal of duplicates using Endnote® [21], 1157 articles were identified. After abstract screening, 119 studies were selected. After full text reading, 64 articles were selected, plus 5 other articles found among the related ones found by additional manual search (Fig. 3). The main reason for excluding articles was a content not addressing AM models issues. Many excluded articles dealt with analogic models, virtual models or perfusion-based models, or related to the orthopedic and veterinary fields. Six articles were not written in English/French/Italian.

Eight articles were excluded after reviewers’ discussion: three authors involved commercial models [22–24], one article dealt with papercraft models [25], one article dealt with a silicon model molded on a 3D printed mold [26] and 3 articles dealt with other surgical fields (orthopedics [27, 28] and veterinary sciences [29]).

**Fig. 4:**
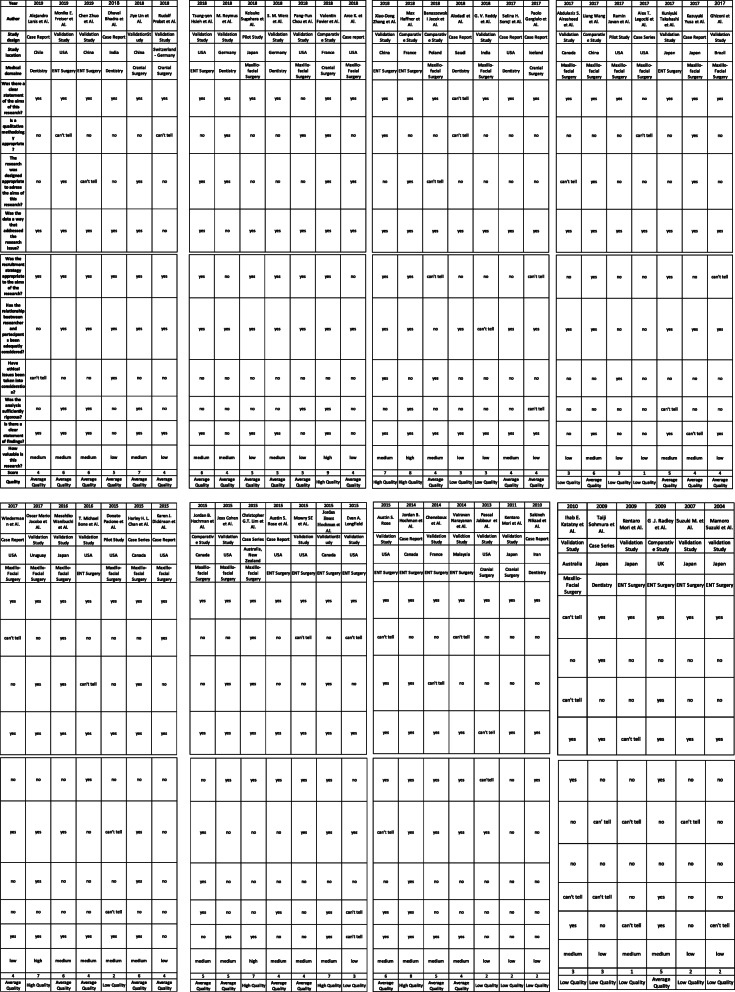
Mesh keyword co-occurrence networks among the retrieved articles. The size of each node is proportionate to its degree and the thickness of the links represents the tie strength

The spatial representation of the relationships between the Mesh keywords was displayed through a science map (Fig. 4). The networks noticeably highlighted the interest for modelling human patients from imaging data through a computer assisted procedure. Bone surgery, prosthetic rehabilitation and radiotherapy were among the most represented families of clinical applications. “Mandible” was the most co-occurring bone keywords. “Material testing”, “treatment outcome” and “clinical competence” were often studied. These graphic references did not show any Mesh keywords regarding the 3D printing technologies themselves (under the threshold).

### Quality assessment (Table 1)

With the modified Critical Appraisals Skills Programme (CASP) tool [20] the reviewers have identified as “high-quality studies” only 8 original researches. The authors classified as “average quality studies” and “poor quality studies” 46 and 15 articles respectively. The lack of quantitative or rigorous tests and appropriate study designs were found in most of the selected studies.

**Table 1:** CASP questions and results of the quality assessment

CASP Questions	Results
Was there a clear statement of the aims of this research?	High Quality
Is a qualitative methodology appropriate?	8 Articles
The research was designed appropriate to address the aims of this research?	
Was the recruitment strategy appropriate tothe aims of the research?	Average Quality
Was the data collected in a way that adressed the research issue?	46 Articles
Has the relationship between researcher and participants been adequately considered?	
Have ethical issues been taken into consideration?	Low Quality
Was the data analysis sufficiently rigorous?	15 Articles
Is there a clear statement of findings?	
How valuable is the research?	

### General information (Table 2)

Only 5 articles (6%) were published before 2008. Ten articles (18%) were published between 2009 and 2014. Forty-two articles (78%) were published between 2015 and March 10, 2019.

**Table 2:** General Information of the included studies

Year	Author	Study Design	Country	Domain	Treatment	Target
2019	Lanis A et al. [30]	Case Report	Chile	Dentistry	Implant treatment	Planning
2019	Freiser ME et al. [31]	Validation Study	USA	ENT Surgery	Temporal bone access	Planning, Simulation, Training
2019	Zhuo C et al. [32]	Validation Study	China	ENT Surgery	Endoscopic sinus surgery	Training, Simulation
2018	Bhadra D et al. [33]	Case Report	India	Dentistry	Endodontic retreatment	Planning
2018	Lin B et al. [34]	Validation Study	China	Cranial Surgery	Tumor removal surgery	Planning, Simulation, Training
2018	Probst R et al. [10]	Validation Study	Switzerland - Germany	Cranial Surgery	Temporal pediatric surgery, coclear implantation	Training
2018	Hsieh TY et al. [9]	Validation Study	USA	ENT Surgery	Endoscopic skull base surgery	Planning, Simulation, Training
2018	Reymus M et al. [35]	Validation Study	Germany	Dentistry	Dental traumatology	Training,
2018	Sugahara K et al. [36]	Pilot Study	Japan	Maxillofacial Surgery	Reconstruction and orthognathic surgery	Planning
2018	Werz SM et al. [37]	Validation Study	Germany	Dentistry	General dentistry	Training
2018	Chou PY et al. [16]	Validation Study	USA	Maxillofacial Surgery	Cleft lip and palate surgery	Simulation
2018	Arce K et al. [15]	Case Report	USA	Maxillofacial Surgery	Mandibular reconstruction	Planning
2018	Lin WJ et al. [38]	Validation Study	China	ENT Surgery	Sinus and skull base anatomical study	Training
2018	Haffner M et al. [39]	Comparative Study	USA	ENT Surgery	Mastoidectomy	Training
2018	Jacek B et al. [40]	Comparative Study	Poland	Maxillofacial Surgery	Mandibular reconstruction with bony free flap	Planning
2018	Alodadi A et al. [41]	Case Report	Saudi	Dentistry	Implantology	Planning
2018	Reddy GV et al. [42]	Validation Study	India	Maxillofacial Surgery	Orthognathic surgery	Training
2017	Favier V et al. [43]	Comparative Study	France	Cranial Surgery	Skull base endoscopic surgery	Planning, Simulation, Training
2017	Somji SH et al. [12]	Case Report	USA	Dentistry	Sinus augmentation	Planning, Simulation, Training
2017	Gargiulo P et al. [19]	Case Report	Iceland	Cranial Surgery	Tumor removal surgery	Planning, Simulation
2017	Alrasheed AS et al. [44]	Validation Study	Canada	Maxillofacial Surgery	Endoscopic sinus surgery	Training
2017	Wang L et al. [45]	Comparative Study	China	Maxillofacial Surgery	Aneurysm surgery	Planning, Simulation, Training
2017	Javan R et al. [46]	Pilot Study	USA	Maxillofacial Surgery	Cranial nerve anatomy	Training
2017	Legocki AT et al. [47]	Case Series	USA	Maxillofacial Surgery	Craniofacial reconstruction	Planning, Simulation, Training
2017	Takahashi K et al. [11]	Validation Study	Japan	ENT Surgery	Temporal bone dissection	Training
2017	Yusa K et al. [18]	Case Report	Japan	Maxillofacial Surgery	Tumor removal	Planning, Simulation
2017	Ghizoni E et al. [48]	Validation Study	Brazil	Maxillofacial Surgery	Craniostenosis	Training
2017	Wiedermann JP et al. [49]	Case Report	USA	Maxillofacial Surgery	Cranio-cervicofacial teratoma	Planning
2017	Oscar Mario Jacobo et Al. [50]	Validation Study	Uruguay	Maxillofacial Surgery	Mandible and orbita recostruction	Planning, Training
2016	Wanibuchi M et al. [51]	Validation Study	Japan	Maxillofacial Surgery	Mastoidectomy	Training
2016	Bone TM et al. [52]	Validation Study	USA	ENT Surgery	Temporal bone surgery	Training
2016	Florentino VGB et Al. [53]	Case Report	Brazil	Maxillofacial Surger	Reconstruction of temporal bone	Planning
2016	Kondo K et Al. [54]	Validation Study	Japan	Cranial Surgery	Craniotomy	Training, Simulation
2016	Lim SH et Al. [55]	Validation Study	Korea	Macillo-Facial Surgery	Mandible reconstruction	Planning
2015	Pacione D et al. [56]	Pilot Study	USA	Maxillofacial Surgery	Deformity of the skull base and craniovertebral junction	Planning
2015	Chan HHL et al. [57]	Case Series	Canada	Maxillofacial Surgery	Head and neck surgery	Training, Simulation
2015	Dickinson KJ et al. [58]	Case Report	USA	Maxillofacial Surgery	Endoscopic resection in esophagus	Planning
2015	Hochman JB et al. [59]	Comparative Study	Canada	Maxillofacial Surgery	Mastoidectomy and skull base surgery	Training
2015	Cohen J et al. [60]	Validation Study	USA	Maxillofacial Surgery	Mastoidectomy	Training
2015	Lim C et al. [17]	Case Series	Australia - New Zealand	Maxillofacial Surgery	Orbital reconstruction	Planning
2015	Rose AS et al. [61]	Case Report	USA	ENT Surgery	Mastoidectomy	Planning, Simulation
2015	Ernoult C. et Al. [62]	Case Series	France	Maxillofacial Surgery	Reconstructive surgery	Simulation
2015	Mowry SE. et al. [63]	Validation Study	USA	ENT Surgery	Temporal bone access	Training
2015	Hochman JB et al. [64]	Validation Study	Canada	ENT Surgery	Temporal bone surgery	Training
2015	Longfield EA et al. [65]	Validation Study	USA	ENT Surgery	Temporal pediatric surgery	Training
2015	Rose AS et al. [66]	Validation Study	USA	ENT Surgery	Temporal bone surgery	Training
2014	Hochman JB et al. [67]	Case Report	Canada	ENT Surgery	Temporal bone surgery	Training
2014	Chenebaux M et al. [68]	Validation Study	France	ENT Surgery	Temporal bone surgery	Training
2014	Narayanan V et al. [69]	Validation Study	Malaysia	ENT Surgery	Skull base surgery	Training
2014	Cui J et al. [70]	Validation Study	China	Maxillofacial Surgery	Cranial trauma	Planning
2014	Gil RS et al. [71]	Validation Study	Spain	Maxillofacial Surgery	Mandible reconstruction	Planning
2014	Jardini AL et al. [72]	Case Report	Brasil	Cranial Surgery	Cranial reconstruction	Planning
2013	Jabbour P et al. [73]	Validation Study	USA	Cranial Surgery	Presigmoid access	Training
2013	Li J et al. [74]	Case Series	China	Maxillofacial Surgery	Orbital reconstruction	Planning
2012	Ciocca L et al. [75]	Case Report	Italy	Maxillofacial Surgery	Mandible reconstruction	Planning
2011	Mori K et al. [76]	Validation Study	Japan	Cranial Surgery	Cerebral revascularization via skull approaches	Training, Simulation
2011	Morrison D et al. [77]	Case Report	Australia	Cranial Surgery	Cranial reconstruction	Planning
2010	Nikzad S et al. [78]	Case Report	Iran	Dentistry	Sinus lift and implant treatment	Planning
2010	Katatny IE et al. [79]	Validation Study	Australia	Maxillofacial Surgery	Mandibular surgery	Planning
2010	Lambrecht JTH et al. [80]	Case Series	Switzerland	Dentistry	Oral surgery	Training
2009	Sohmura T et al. [81]	Case Series	Japan	Dentistry	Implant treatment	Planning, Training
2009	Mori K et al. [82]	Validation Study	Japan	ENT Surgery	Skull base surgery	Training
2009	Radley GJ et al. [83]	Comparative	UK	ENT Surgery	Endoscopic sinus surgery	Training
2009	Cohen A et al. [84]	Case Series	Syria	Maxillofacial Surgery	Mandible reconstruction	Planning
2007	Suzuki M et al. [85]	Validation Study	Japan	ENT Surgery	Temporal bone surgery	Training
2007	Mavili ME et al. [86]	Case Series	Turkey	Dentistry	Orthognatic surgery	Planning, Simulation
2004	Suzuki M et al. [87]	Validation Study	Japan	ENT Surgery	Temporal bone access	Training
2003	Muller A et al. [88]	Case Series	Germany	Cranial Surgery	Cranioplasty, tumor removal	Planning, Simulation
1997	Löpponen H et al. [89]	Case Report	Finland	ENT Surgery	Cochlear implant	Simulation, Training

Among all articles, 3 were pilot studies, 12 were case reports, 4 were case series, 29 were validation studies and only 6 were comparative studies. The authors classified as “validation studies” all original researches that had described and eventually evaluated a process to fabricate a printed bone model. The reviewers classified as “comparative studies” all the researches that compared models printed by different techniques or models printed by different printers using the same technique. Lastly, a paper comparing a bone model to a cadaver’s bone was also considered as a comparative study [59].

### Purposes of the articles

The models mentioned in the selected articles were used for different purposes (Table 2). In 31 articles the models were used to plan a surgery, in 19 they were used to simulate the surgery, and in 32 they were used for training of students or clinicians. This total (82) exceeded the total number of papers, as some models were used for multiple purposes.

### Surgical field

The reports on 3DP models concerned multiple surgical domains (Table 2). Oral and maxillofacial surgery had the largest share with 43% of articles describing the use of AM models, followed by ENT surgery (29%), dentistry (14%), and cranial surgery (14%).

### Therapy (Table 2)

In dentistry, bone models were more frequently used for simulating dental implant placement [30, 41, 81]. In oral and maxillofacial surgery, the models were more frequently used for planning a bone reconstruction [15–17, 36, 40, 47, 50, 53, 72] or a tumor removal [18, 49, 56]. The models prepared for ENT surgery were mostly used for training surgical temporal access [10, 31, 52, 63–68, 85, 87] and mastoidectomy [39, 51, 59–61]. Finally, in the field of cranial surgery, the models were most frequently used for the training of the pre-sigmoid approach [73] or craniotomy [54].

### Image acquisition and processing

Image acquisition and processing are the first steps to create a 3DP model (Table 3). The most frequently used radiological exam was the CT, followed by cone beam computed tomography (CBCT) and MRI. Software was used to process the radiological data. The most frequently used were Mimics® (Materialise, Leuven, Belgium), followed by OsiriX® (Pixmeo, Geneva, Switzerland) and 3D Slicer® (Surgical Planning Laboratory, Isomics Incorporated, Cambridge, USA). Most of the authors did not specify entirely their digital work-flow to create the STL printable file, making it difficult to reproduce the procedure properly.

**Table 3:** Workflow’s analysis of the included studies

Year	Author	Data Acquisition	Images Processing Software	Printing Technique	Material
2019	Lanis A et al. [30]	CBCT	CoDiagnostiX	Vat photopolymerization	Photosentitive Resin
2019	Freiser ME et al. [31]	CT	3D Slicer	Vat photopolymerization	Photosentitive Resin
2019	Zhuo C et al. [32]	CT	Mimics	Material Extrusion	PLA
2018	Bhadra D et al. [33]	CBCT	–	Material Extrusion	–
2018	Lin B et al. [34]	CT	Mimics	Material Jetting	Photosensitive Resin
2018	Probst R et al. [10]	μCT	–	Binder Jetting	Cast Powder and Bonding Agent
2018	Hsieh TY et al. [9]	CT	–	Material Jetting	Photosensitive Resin
2018	Reymus M et al. [35]	CBCT	InVesalius	Vat photopolymerization	Photosensitive Resin
2018	Sugahara K et al. [36]	CT	Mimics	Material Jetting	Photosensitive Resin
2018	Werz SM et al. [37]	CT	3D Slicer	Material Extrusion	PLA, ABS
2018	Chou PY et al. [16]	CT	Mimics	Material Extrusion	ABS
2018	Arce K et al. [15]	CT	Mimics	Vat photopolymerization	Photosentive Resin
2018	Lin WJ et al. [38]	CT	Mimics	Material Extrusion	PLA
2018	Haffner M et al. [39]	CT	Slicer	Material Extrusion	PLA, ABS, Nylon, PETG, PC
2018	Jacek B et al. [40]	CT	Slicer	Material Extrusion	ABS
2018	Alodadi A et al. [41]	CBCT	–	–	–
2018	Reddy GV et al. [42]	–	–	–	–
2017	Favier V et al. [43]	CT	Medical Image Segmentation Tool	Binder Jetting, Material Jetting, Powder Bed Fusion, Material Extrusion	Calcium Sulfate Hemihydrate, Opaque Resin, Polyamide, Polycarbonate
2017	Somji SH et al. [12]	CBCT	OsiriX	Vat photopolymerization	Photosensitive Resin
2017	Gargiulo P et al. [19]	CT and MRI	Mimics	Material Extrusion	ABS
2017	Alrasheed AS et al. [44]	CT	Mimics	Material Jetting	Photosensitive Resin
2017	Wang L et al. [45]	CTA	Mimics	Material Jetting	Photosensitive Resin
2017	Javan R et al. [46]	MRI	OsiriX	Powder Bed Fusion	Polyamide
2017	Legocki AT et al. [47]	–	OsiriX	Material Extrusion	PLA
2017	Takahashi K et al. [11]	CT	ZedView	Binder Jetting	Plastic Powder and Colored Binders
2017	Yusa K et al. [18]	CT and MRI	ZedView	Binder Jetting	Composite Powder
2017	Ghizoni E et al. [48]	CT	Mimics	Powder Bed Fusion	Polyamide
2017	Wiedermann JP et al. [49]	CT and MRI	–	–	–
2017	Oscar Mario Jacobo et Al. [50]	CT	–	Material Extrusion	PLA
2016	Wanibuchi M et al. [51]	–	–	Powder Bed Fusion	Polyamide and Glass Fiber
2016	Bone TM et al. [52]	CT	OsiriX	Material Extrusion	ABS
2016	Florentino VGB et Al. [53]	CT	InVesalius	–	
2016	Kondo K et Al. [54]	CT and MRI	–	Binder Jetting	Calcium sulfate hydrate
2016	Lim SH et Al. [55]	CT	Mimics	Material Jetting	–
2015	Pacione D et al. [56]	CT	Philips Intellispace Portal	Material Jetting	Photosensitive Resin
2015	Chan HHL et al. [57]	CT	Mimics	Material Extrusion	ABS, ABS and Powder, Polycarbonate
2015	Dickinson KJ et al. [58]	CT and MRI	Mimics	Material Jetting	Photosensitive Resin
2015	Hochman JB et al. [59]	CT	Mimics	Binder Jetting	Composite Powder
2015	Cohen J et al. [60]	CT	ITK-Snap	Material Extrusion	ABS
2015	Lim C et al. [17]	CT	–	Material Extrusion	–
2015	Rose AS et al. [61]	CT	Mimics	Material Jetting	Photosensitive Resin
2015	Ernoult C. et Al. [62]	–	OsiriX	Material Extrusion	ABS
2015	Mowry SE. et al. [63]	CT	OsiriX	Material Extrusion	ABS
2015	Hochman JB et al. [64]	μCT	Mimics	–	–
2015	Longfield EA et al. [65]	CT	–	Binder Jetting	–
2015	Rose AS et al. [66]	CT	Mimics	Material Jetting	Photosensitive Resin
2014	Hochman JB et al. [67]	CT	Mimics	Binder Jetting	Composite Powder and Colored Binders
2014	Chenebaux M et al. [68]	CT	Magics	Vat photopolymerization	Photosensitive Resin
2014	Narayanan V et al. [69]	CT and MRI	Biomodroid	Material Jetting	Photosentive Resin
2014	Cui J et al. [70]	CT	Materialise	Powder Bed Fusion	Polystirene
2014	Gil RS et al. [71]	CT	Materialise	Vat photopolymerization, Powder Bed Fusion	–
2014	Jardini AL et al. [72]	CT	InVesalius	Binder Jetting	–
2013	Jabbour P et al. [73]	–	–	–	–
2013	Li J et al. [74]	CT	3DMSR	Powder Bed Fusion	Polystirene
2012	Ciocca L et al. [75]	CT	CFT	Material Extrusion	ABS
2011	Mori K et al. [76]	–	–	Powder Bed Fusion	Polyamide
2011	Morrison D et al. [77]	CT	Mimics	Material Extrusion	ABS
2010	Nikzad S et al. [78]	CT	Simplant	Binder Jetting	Polyamide
2010	Katatny IE et al. [79]	CT	InVesalius	Material Extrusion	ABS
2010	Lambrecht JTH et al. [80]	CBCT	Magics	Material Jetting	–
2009	Sohmura T et al. [81]	CT	VGStudio Max	Material Extrusion	ABS
2009	Mori K et al. [82]	CT	–	Powder Bed Fusion	Polyamide
2009	Radley GJ et al. [83]	μCT	Mimics	Powder Bed Fusion	Polyamide
2009	Cohen A et al. [84]	CT	Magics	Material Jetting	–
2007	Suzuki M et al. [85]	CT	–	Vat photopolymerization	–
2007	Mavili ME et al. [86]	CT	Mimics	Material Jetting	–
2004	Suzuki M et al. [87]	CT	–	Powder Bed Fusion	Polyamide
2003	Muller A et al. [88]	CT	–	Vat photopolymerization	Photosensitive resin
1997	Löpponen H et al. [89]	CT	–	Vat photopolymerization	Photosensitive resin

### Printing systems and materials

Material extrusion (ME) printing was the preferred technique to create models, followed by material jetting (MJ), respectively 32% and 22% of the articles (Table 3). Binder jetting and vat photopolymerization (VP) technique were both involved in 10% of papers. Powder bed fusion printers were used in 16% of articles while 9% did not mentioned the type of 3D printer involved. Among all the references selected, the most frequently used material was acrylonitrile butadiene styrene (ABS), currently only used with material extrusion printers.

### Quantitative evaluation

Accuracy and mechanical characteristics are strongly dependent on the 3D printer’s characteristics, on the involved printer material and the size of the model. Only three authors analyzed the mechanical properties of their models through quantitative tests [43, 67, 83]. Due to the differences in the printing materials and measuring methods, it was not possible to compare their results (Table 4). For the same reason, it is impossible to compare the model accuracy, despite the fact that several authors measured the geometric discrepancy (Table 5) [9, 43, 51, 61, 79].

**Table 4:** Quantitative evaluations of 3DP models’ mechanical properties

Year	Author	Objective	Methods	Results
2017	Favier V et al. [43]	Evaluation of several consumer-grade materials for creating patient-specific 3D-printed skull base model for anatomical learning and surgical training.	Four different materials were compared to fabricate the models Force sensors were used to evaluate: - Average force needed to break thin walls with the surgical suction tip - Energy spent and reported instantaneous forces during a 6 mm depth drill	All materials displayed higher mechanical properties than human cadaver bone Resin and PA were not adapted because forces exceeded to break thin walls were too high (200 N). Using “Multicolor” and PC, the forces applied were 1.6 to 2.5 / 3.5 times higher than bone. Energy spent during drilling was respectively 1.6 and 2.6 times higher on bone than on PC and Multicolor. Finally, PC and Multicolor were the more adapted materials for this application.
2014	Hochman JB et al. [67]	To generate a rapid-prototyped temporal bone model from computed tomography (CT) data with a specific focus on internal anatomic fidelity.	Three point bending tests, using a Texture Analyzer® were performed to determine the elastic modulus and yield point. Thanks to a 3-axis accelerometer the drill vibration during the drilling was evaluated on different materials.	The printed bone models were highly realistic. Void space representation was excellent with 88% concordance between cadaveric bone and the resultant rapid-prototyped temporal bone model. Ultimately, cyanoacrylate with hydroquinone was determined to be the most appropriate infiltrant for both cortical and trabecular simulation. The mechanical properties of all tested infiltrants were similar to real bone
2009	Radley GJ et al. [83]	To fabricate and characterize human sinus phantoms by 3D printing for surgery simulation	A modified surgical instrument was used to evaluate the necessary force to break thin walls made by test materials compared to cadaver bone.	The materials that could be successfully combined into a suitable fluid were polyurethanes, polishes, and suspended cellulose/polyesters (hardeners).

**Table 5:** Studies including a quantitative evaluation of 3DP models’ accuracy

Year	Author	Objective	Methods	Results / Conclusions
2018	Hsieh TY et al. [9]	Fabrication of sinus and skull base 3D-printed models for endoscopic skull base surgery	Numerical measurements and image navigation were used to localize several landmarks on the CT images of the patients compared to the CT of the 3DP model. Evaluation of the surgeons perceptions (Likert scale) after dissecting printed models (Haptic Feedback and anatomical accuracy)	Comparisons demonstrated less than 5% difference between the images. Lickert scores were positive for haptic feedback (4,67/5) and anatomical accuracy (4/5)
2017	Favier V et al. [43]	Evaluation of several consumer-grade materials for creating patient-specific 3D-printed skull base model for anatomical learning and surgical training.	4 different printing materials were compared for accuracy, surgical forces needed to break and drill thin walls	PC and PA displayed the highest printing accuracy. The use of printed models in PC is a good substitute to human cadaver bone for skull base surgery simulation
2017	Legocki AT et al. [47]	Evaluation of the feasibility of using low-cost 3D printers for the fabrication of anatomical models for craniofacial reconstruction	Comparison of in-house printing process of surgical models vs commercial printed models. 3 different mandible models Analogical measurements with digital caliper + other criteria (cost, production speed, sterilization …)	Similar results for the accuracy of both techniques Nerve canal visibility, tooth root visibility, and sterilizability were inferior for in house models Overall, the in-house technique is adapted for education and surgical planning, including preoperative plates bending.
2016	Wanibuchi M et al. [51]	Fabrication of a 3D temporal bone model and validation of accuracy	Accuracy was investigated by fusion of the original CT of patient’s temporal bone and the 3DP model’s CT	The differences between both CT images were below 1 mm The printed models are adapted for surgical training.
2015	Rose AS et al. [61]	Producing a patient-specific model for pre-operative simulation in pediatric otologic surgery	Case report of cholesteatoma Measurement and comparison of distances between several anatomic landmarks (CT scan / Printed model / During surgery)	The variability was minimal, in terms of absolute distance (mm) and relative distance (%), in measurements between anatomic landmarks obtained from the patient intra-operatively, the pre-operative CT scan and the 3D-printed models.
2010	Katatny IE et al. [79]	Simulation of shape and CT values of pulmonary parenchyma and lesions of various sizes using 3DP	Comparison of patient original CT and printed model CT	High accuracy was observed Patient-specific CT imaging phantoms can be obtained by FDM printer It can be used for the calibration of CT intensity and validation of image quantification software.

### Qualitative evaluation

Most of the authors analyzed the accuracy and haptic feedback of their models using self-made questionnaires and they usually concluded that 3DP models were accurate. Two authors declared respectively less than 100 and 125 μm of geometrical discrepancy between the real bone and the model [33, 81]. Some other authors stated that discrepancies could reach 680 μm, depending on the size of the model [47]. The haptic feedback was declared adequate in 75% of articles that investigated it through qualitative tests. A model made of calcium sulfate hemihydrate was considered “too hard” [43] and another 3DP model was “too soft” [65]. Few authors showed that it was difficult to print small bones [10, 52, 63] and it was reported that some materials melted during drilling [37, 43]. Few authors reported benefiting of a reduction in treatment time up to 20% in the operating room thanks to the models [18, 50, 75]. A third (35%) of the articles affirmed that the clinical outcomes could potentially be improved by using these techniques, thanks to the better planning and the enhanced comprehension of the patient pathological status. Considering costs and production time, ME printed models were the cheapest [38, 39, 42, 60] and the fastest printers [65, 67]. Cost-effectiveness depended on each clinical case and was more striking when several models needed [82]. Only 7% of the authors suggested that their method was not cost-effective [43, 61, 63, 78].

## Discussion

Our original impression was validated by the results, suggesting that, before investing in a printer, the major application of the models needs to be considered. Oral and maxillofacial models for bone surgery applications are mostly used for planning and simulating surgical interventions. Printed products exhibit a wide range of different properties, varying with the machine and the printing material. The main results showed that surgical treatment times can be reduced up to 20%, and that the failure rate tends to decrease [40, 50, 71]. Many authors suggested that clinical outcomes can be improved, but their findings were not supported by any control group [12, 18, 19, 33–36, 41, 42, 50, 56, 58, 76, 78, 86]. Only Banaszewski et al. involved a control group to compare the use of the 3D printed model for planning the surgical reconstruction of the mandible to the traditional technique. They found that the functional and aesthetic results were greater in the group where a 3DP models were applied [41]. A planning model needs to be accurate, but cheap also, as one patient cannot cover extensively all expenses. A training model requires essentially to reproduce relevant haptic feedback and to be an inexpensive investment. These two qualities are also expected to simulate a surgical intervention, but also with a high level of accuracy.

### Accuracy

The MJ printers are currently the most accurate printers, with printed models exhibiting a geometrical discrepancy of 90 μm when compared to the patient’s bone [76]. The second most accurate printing technique, according to the analyzed articles, is powder bed fusion (PBF). Wanibuchi et al. showed an accuracy ranging from 100 μm to 300 μm on a temporal bone model measured with a digital caliper [51]. This result was confirmed by another study where a geometrical discrepancy of 150 μm was observed between the model and the bone [43]. BJ and ME were reported to be less accurate methods. A geometrical discrepancy of 400 μm was observed when using a BJ printer to print a skull base [43]. Most of the researches involving ME printers did not measure quantitatively the models’ accuracy, except in one case where they reported a discrepancy reaching 680μm [47]. Our study did not retrieve any paper measuring the accuracy of VP printers, but was previously reported as being high [8].

The lack of quantitative evaluation of the printing accuracy was one of the major limitations of the studies included in this review. The accuracy is related to the printer, the radiological image segmentation process, the size of the printed object and the printing material. For example, a ME printer cannot reach the same precision as an VP or MJ printer due for first to the dimension of the nozzle, but its accuracy could be sufficient to reach the operator’s purposes. Depending on the radiological images processing technique a 3D-printed model will always exhibit some discrepancies, the operator has to keep it in mind processing the radiological data.

### Haptic feedback

A good haptic feedback is the most important characteristic of training models and it is strongly dependent on the mechanical characteristics of the printing material. The two fundamental parameters for a model that aims to reproduce the bone haptic feedback are adequate elastic modulus and tensile strength. No quantitative test is currently available to describe the surgeon’s haptic feedback during a surgical intervention. Most of the authors created their own questionnaires and asked students and surgeons with different experience about their sensations. Thus, the results were difficult to compare as evaluation protocols were different and also because of the conclusions subjective. However, most of the them were satisfied with their printed models.

The principal materials for ME printers were polylactic acid (PLA), ABS, polycarbonate (PC), polyethylene terephthalate glycol-modified (PETG) and nylon. In the articles included in this review, there was no consensus regarding the best material to reproduce the bone characteristics. One of the PLA’s advantages was its biological properties, as it is known to be biodegradable and non-toxic. Moreover, its haptic feedback was similar to bone at low temperature while drilling [37]. Haffner et al., compared five different materials, and stated that PETG was the most realistic material, followed by PC, PLA and ABS. Nylon properties were considered as not realistic enough [39]. PC was blamed to melt too easily during drilling while ABS could easily reproduce the bone haptic feedback during a cortical mastoidectomy [43]. Favier et al. compared the mechanical characteristics of their models. With Young’s Modulus respectively of 2000–3000 N/mm2 and 1700 N/mm2, the MJ and PBF printed models were considered as realistic. Regarding PBF printing, Mori et al. reported that their model was realistic but the feeling of drilling the cancellous part of the bone model lacked the ‘crispy touch’ of real bone [82]. This subjective declaration underlines the need for objective criteria to evaluate the haptic feedback of the models. Among all the materials used in the BJ printing technique, cyanoacrylate powder with hydroquinone resembled the most to sheep cortical bone, which was often used as a surgical training model [85].

Unfortunately, no author did quantitative or comparative tests using models printed by VP. However most authors suggested that this technique was efficient for creating adequate models, enhancing the quality of the training [12, 35] and suitable for planning complex surgeries [15] or dental implant treatments [30].

The segmentation technique is also an important parameter that it has to be considered for obtaining realistic 3D printed models. Segmenting the trabecular bone structures results in a more realistic haptic feedback when compared to fully solid prints.

### Cost

Cost remains an important parameter that cannot be ignored. There are multiple additional costs in terms of software, printers, printing materials, operators and training hours to produce an in-house 3DP model.

In order to print a model, the first step is to process the DICOM file into an STL file. Among all the commercial software available, the commercial software package Mimics®, was the most widely used software, despite a relatively high cost when compared to others. Many free or open source software are available, like ITK-Snap®, Slicer® or InVesalius® [31, 35, 39, 40, 60, 79]. OsiriX® possesses a free version (demo) that allows to export STL renderings [12, 46, 47, 52, 63].

ME printers were the cheapest printers, with reported prices ranging between 2500$ and 3000$ [47, 82]. However, as previously mentioned, these showed limitations in terms of accuracy. PLA and ABS were the cheapest reported materials [32, 37, 39, 47, 52, 82]. PC cost was reported to range from 105$ to 155$ for the production of a mandible [43, 57]. The cost for a temporal bone model using BJ was around 400$ [67]. MJ models costs ranged from 270$ to 1000$. One team affirmed that these models were too expensive [66], but two others suggested that they were satisfied by their investment in these models [15, 56]. LS printers are not cheap, but no authors talked mentioned any price. Printing a skull base using polyamide with an LS printer was reported to cost 250–280$ [85]. Two teams used VP printers and they declared being satisfied by the results [12, 35]. A forgotten cost is related to the post-curing machine for object printed trough VP technologies.

The production time can also be considered as a decisive cost. ME printers were the fastest, producing a pediatric temporal bone model in 4.5 h and a mandible with a maxilla in 6 h [37, 39]. The production time of an MJ model was less than a day [9, 56]. The time needed to design the STL file is also important to consider. Only one author discussed about the total production time of a LS printer, and reported a need for 4 to 5 days [43]. The learning curve of a few months to master and to properly use the software for STL processing is obvious. In every case, the conversion from the DICOM to an adequate STL file could take several hours. One author reported that “the 3DP technique is really cost-effective, only if the operator plans to produce several models to amortize the cost of the 3D printer” [82].

It’s important to know these costs because the cost-benefit ratio has to be considered before investing in this technique that can get several advantages but it presents some drawbacks in comparison to traditional techniques [63, 78].

### Suggestions

As pre-surgical tools, 3D models can make the surgical outcomes more predictable and safer, reducing the surgeon’s stress and the intervention time [40, 50]. Furthermore, they can strongly improve the quality of clinical education, allowing students to simulate various surgical interventions and to discuss easily about their clinical cases with their mentors [34, 35, 42].

Evaluating the best image processing workflow remains difficult as no author described neither the entire workflow not the human cost involved. The most used training models are made with ME printers. This technology is the cheapest and allows producing suitable training models, despite their limited accuracy. The most appropriate ME printing materials are ABS, PLA and PETG [43]. Temporal bone models printed with PETG were reported to ensure adequate haptic feedback whileperfor drilling, and they were very helpful during training sessions [39].

To obtain an adequate simulation model, BJ and VP printers seem more indicated. They showed adequate performances in reproducing training models of the maxilla and their accuracy is really satisfying for creating planning models [12, 18]. It is also possible to create models for simulating surgeries, thanks to their good haptic feedback [12, 14, 18]. Also MJ printers allow to produce models that provide good haptic feedback and that can be used to simulate complex surgeries before entering the operating room [14]. Generally, they cost more than the VP ones, as well as the BJ ones [35, 61]. PBF printers allow to produce accurate bone models in polyamide and glass fiber, but without satisfying haptic feedback [82].

**Fig. 5:**
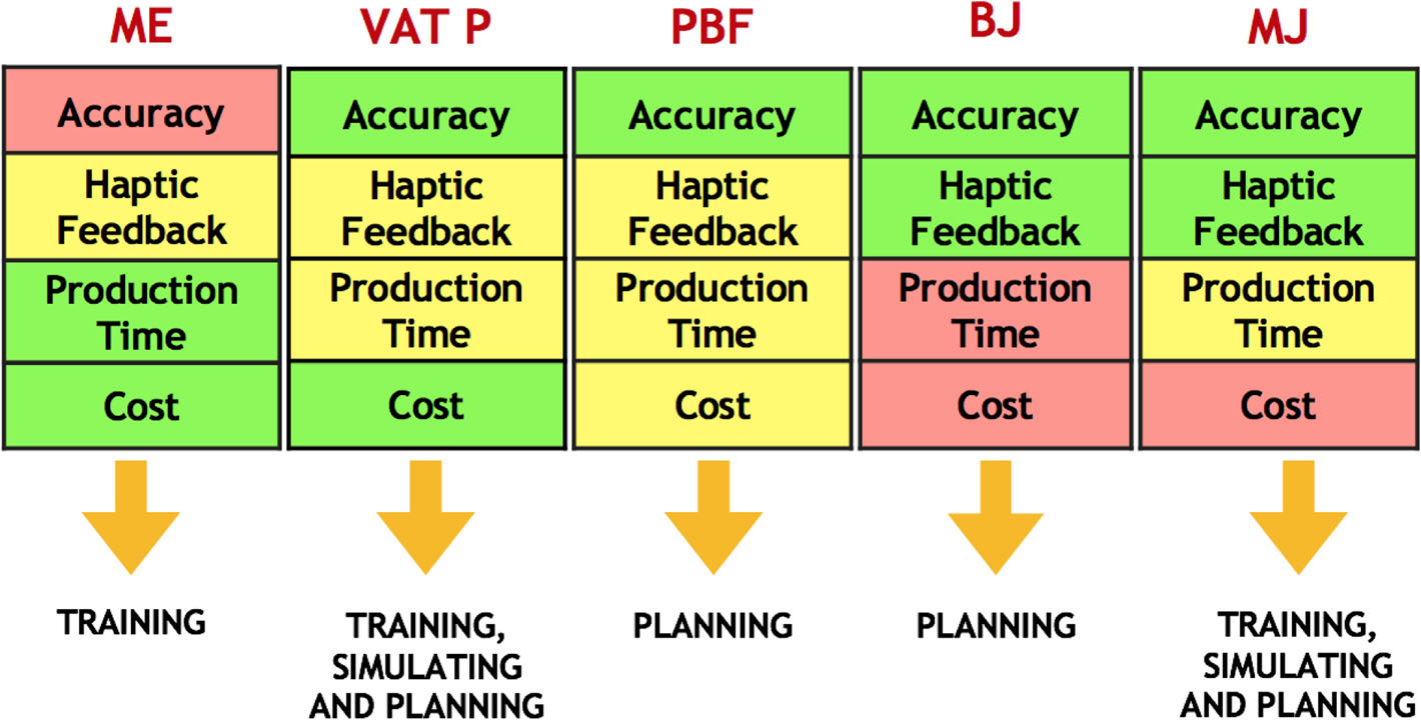
Advantages (green) and limitations (red) of different 3D printing technology to create 3D anatomical models. These characteristics lead the choice for the best 3D printer technique for every clinical or academical purpose

Printing time and cost are very variable, depending directly on the type of 3D printer, the printing material, the accuracy and the mechanical characteristics required. Figure 5 demonstrates the main differences among the analyzed 3D printing technologies, depending on the applications.

## Conclusions

The present literature review showed that nowadays, AM models are useful tools in the surgical field.

Several parameters must be considered before choosing a 3D printing model workflow, such as the processing software, the type of 3D printer, the expected mechanical characteristics, accuracy and haptic feedback of the printing material, the production time and the human and material costs.

Due to the large amount of different parameters that has to be considered by the operator, the financial investment in a 3D printer should be made with the precise idea of the final application.

### Limitations

This analysis was at first limited by the diversity of workflows and applications, involving different materials, printers and testing methods. Despite difficulties for comparing results from a study to another, some common protocols were found for the 3 main purposes of 3D-printed bone models (planning, simulation and training).

The lack of common reliable qualitative tests to evaluate the models was an evident limitation, thus future studies should focus on standardized methods to evaluate 3D-printed models of bone macro- and micro-structures.

## Data Availability

All data generated or analyzed during this study are included in this published article [and its supplementary information files].

## Abbreviations

3D: Three-dimensional
3DP: Three-dimensional printed; Three-dimensional printing
ABS: Acrylonitrile butadiene styrene
AM: Additive manufacturing
BJ: Binder jetting
CAD/CAM: Computer-aided design/computer-aided manufacturing
CBCT: Cone beam computed tomography
CT: Computed tomography
DICOM: Digital imaging and communications in medicine
ENT: Ear, nose and throat
ME: Material extrusion
MJ: Material jetting
MRI: Magnetic resonance imaging
PBF: Powder bed fusion
PC: Polycarbonate
PETG: Polyethylene terephthalate glycol-modified
PLA: Polylactic acid
STL: Standard triangulation language
VP: Vat photopolymerization
