# Correction to: 3D printed bone models in oral and craniomaxillofacial surgery: a systematic review

**DOI:** 10.1186/s41205-020-00088-z

**Published:** 2020-12-02

**Authors:** Matteo Meglioli, Adrien Naveau, Guido Maria Macaluso, Sylvain Catros

**Affiliations:** 1 University Center of DentistryDepartment of Medicine and Surgery, University of Parma, Via Gramsci 14, 43126 Parma, Italy; 2 Department of Prosthodontics, Dental Science Faculty, University of Bordeaux, 46 rue Léo-Saignat, 33076 Bordeaux, France; 3 Dental and Periodontal Rehabilitation Unit, Saint Andre Hospital, Bordeaux University Hospital, 46 rue Léo-Saignat, 33076 Bordeaux, France; 4 Biotis Laboratory, Inserm U1026, University of Bordeaux, 46 rue Léo-Saignat, 33076 Bordeaux, France; 5 IMEM-CNR, Parco Area delle Scienze 37/A, 43124, Parma, Italy; 6 Department of Oral Surgery, UFR d’Odontologie, University of Bordeaux, 46 rue Léo-Saignat, 33076 Bordeaux, France; 7 Service de Chirurgie Orale, CHU de Bordeaux, 46 rue Léo-Saignat, 33076 Bordeaux, France

**Correction to: 3D Print Med (2020) 6:30**

**https://doi.org/10.1186/s41205-020-00082-5**

Following publication of the original article [1], the authors identified an error in Fig. 4 which was introduced during typesetting. The correct figure is given below.

**Fig. 4:**
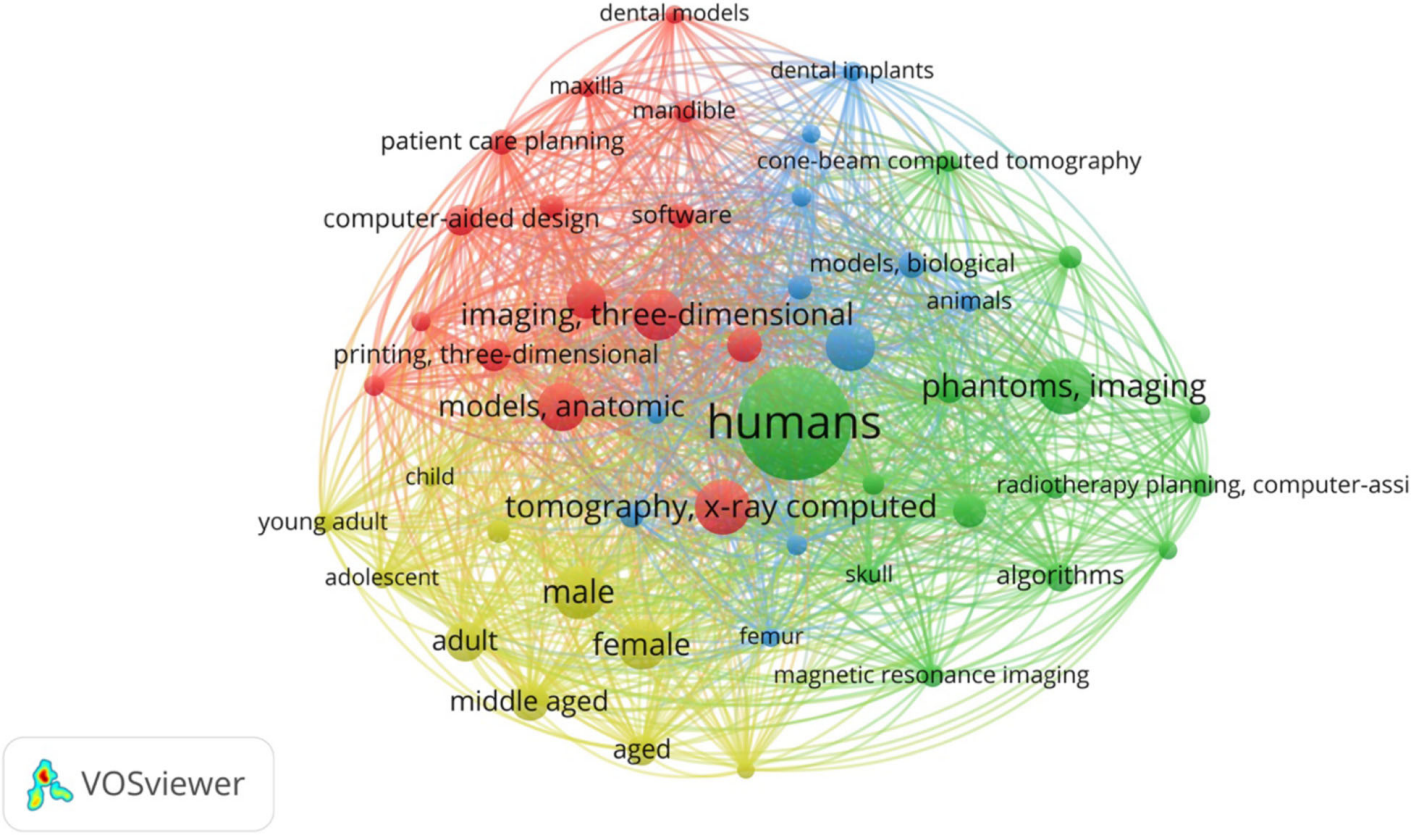
Mesh keyword co-occurrence networks among the retrieved articles. The size of each node is proportionate to its degree and the thickness of the links represents the tie strength

The original publication has been corrected. The publisher apologizes to the readers and authors for the inconvenience.
